# Analysis of somatic retrotransposition in human cancers

**DOI:** 10.1186/1753-6561-6-S6-O23

**Published:** 2012-10-01

**Authors:** Eunjung Lee, Rebecca Iskow, Lixing Yang, Omer Gokcumen, Psalm Haseley, Lovelace J Luquette, Jens G Lohr, Christopher C Harris, Li Ding, Richard K Wilson, David A Wheeler, Richard A Gibbs, Raju Kucherlapati, Charles Lee, Peter V Kharchenko, Peter J Park

**Affiliations:** 1 Center for Biomedical Informatics, Harvard Medical School, Boston, MA 02115, USA; 2 Division of Genetics, Brigham and Women's Hospital, Boston, MA 02115, USA; 3 Department of Pathology, Brigham and Women's Hospital, and Harvard Medical School, Boston, MA 02115, USA; 4 The Eli and Edythe Broad Institute, Cambridge, MA 02412, USA; 5 Dana-Farber Cancer Institute, Boston, MA 02115, USA; 6 The Genome Institute, Washington University, School of Medicine, St Louis, MO 63108, USA; 7 Human Genome Sequencing Center, Baylor College of Medicine, Houston, TX 77030, USA; 8 Department of Genetics, Harvard Medical School, Boston, MA 02115, USA

## Background

Close to half of the human genome is derived from transposable elements (TEs), and some TE families continue to generate new insertions through RNA-mediated mechanisms. Due to its mutagenic potential, such retrotransposition is normally suppressed by epigenetic and post-transcriptional mechanisms. However, the epigenetic and regulatory disruptions commonly observed in cancers may allow for TE activation, and a few examples have been reported in lung and colon cancer previously.

## Materials and methods

To systematically evaluate the frequency of such events across different tumor types and assess their impact in human cancers, we developed Tea (Transposable element analyzer), a computational pipeline to detect TE insertions at single nucleotide level and extract their mechanistic signatures. We applied Tea to the high-coverage (>30×) tumor and matched normal genome pairs from 43 cancer patients across five tumor types as well as three healthy individuals.

## Results

We identified 194 high-confidence somatic TE insertions (183 L1, 10 Alu, 1 ERV), most of which were generated through endonuclease-mediated retrotransposition mechanism. The novel L1 and Alu insertions were all found in the epithelial cancers (colorectal, prostate, ovarian), and none were detected in the examined blood or brain tumor samples. The somatic L1 insertions tend to occur in genes that are commonly mutated in cancer, and disrupt the expression of the targeted genes. To further illustrate the distinct genomic distribution of the somatic TE landing sites, we compared their placement with the 7,449 non-reference polymorphic TE insertions that we have identified from 44 normal genomes. The TE landing sites are strongly biased towards genomic regions that exhibit cancer-specific decrease in DNA methylation.

## Conclusions

Our analysis illustrates the functional impact of somatic TE insertions and suggests resulting positive selection toward tumorigenesis.

